# The association between health literacy and diabetes self-management among adolescents with type 1 diabetes

**DOI:** 10.1186/s12887-026-07244-0

**Published:** 2026-07-15

**Authors:** Afsaneh Ranaei, Mansooreh Abbasi, Hadi Tehrani

**Affiliations:** 1 School of Health, Student Research Committee, Mashhad University of Medical Sciences, Mashhad, Iran; 2 Department of Health Education and Health Promotion, School of Public Health, Shahid Sadoughi University of Medical Sciences, Yazd, Iran; 3 Department of Health Education and Health Promotion, School of Health, Mashhad University of Medical Sciences, Mashhad, Iran; 4 Social Determinants of Health Research Center, Mashhad University of Medical Sciences, Mashhad, Iran

**Keywords:** Health literacy, Diabetes self-management, Diabetes mellitus type 1, Adolescent

## Abstract

**Objective:**

This study examined the association between health literacy and diabetes self-management among adolescents with type 1 diabetes in South Khorasan Province, Eastern Iran.

**Methods:**

This cross-sectional study included 140 adolescents aged 10–18 years with type 1 diabetes, recruited through census sampling from all adolescents aged 10–18 years with type 1 diabetes who had active records in the SIB electronic health system in South Khorasan Province. data were collected using the Health Literacy Measure for Adolescents (HELMA) and the Adolescent Self-Management and Independence in Diabetes (A-SMOD) questionnaires. Mann–Whitney U, Kruskal–Wallis, and Spearman correlation analyses were performed.

**Results:**

The mean health literacy score was 135.79 ± 28.40 (range: 44–220), corresponding to the marginal level per HELMA classification. The mean diabetes self-management score was 133.36 ± 20.22 (range: 48–192). No significant gender differences were observed (*p* > 0.05). Diabetes self-management scores were significantly higher in the 16–18 age group (144.76 ± 18.45; *p* < 0.001). Adolescents diagnosed within the past three years had lower diabetes self-management scores (127.02 ± 18.79; *p* < 0.001). Higher parental education and having a family member with diabetes were associated with better health literacy and diabetes self-management scores (*p* < 0.05). A strong positive correlation was found between health literacy and diabetes self-management (Spearman *r* = 0.76, *p* < 0.001).

**Conclusion:**

Health literacy was at a marginal level and was strongly associated with diabetes self-management in adolescents with type 1 diabetes. Literacy-based educational interventions incorporating family support may improve self-management outcomes in this population.

## Introduction

Type 1 diabetes (T1D) is one of the most common chronic diseases among children and adolescents, affecting more than one million young individuals worldwide. Effective management of this condition requires continuous monitoring and strict glycemic control to prevent long-term complications. T1D results from the destruction of pancreatic beta cells and subsequent insulin deficiency, requiring lifelong insulin therapy and regular blood glucose monitoring [1]. The diagnosis of T1D during childhood presents substantial challenges for both patients and their families and may influence multiple aspects of physical health, psychological well being, and future lifestyle [2].

The global incidence of type 1 diabetes among children under 14 years of age ranges from approximately 1 to 35 cases per 100,000 individuals annually across different countries. In Iran, the reported incidence rate is approximately 3.7 cases per 100,000 children under the age of 14. Worldwide, hundreds of thousands of children are currently living with T1D, and more than 50,000 cases have been reported in Iran [3, 4].

Poor glycemic control may lead to severe complications such as diabetic ketoacidosis, which is associated with significant morbidity and mortality, including neurological and renal complications. Because T1D typically develops early in life, affected individuals may experience both acute and chronic complications over many years if the disease is not properly managed [5, 6]. Effective diabetes management during adolescence has been shown to reduce the risk of vascular and neurological complications by approximately 27–76% [7].

Managing type 1 diabetes during adolescence is particularly challenging. Adolescents often spend more time outside the home, experience reduced parental supervision, and seek greater independence, which may negatively influence adherence to recommended treatment regimens. In addition, adolescents may adopt maladaptive coping strategies such as avoidance or neglect of disease management responsibilities [8]. For this reason, one of the primary goals of healthcare systems is to support adolescents with T1D in developing the necessary skills to effectively manage their condition and maintain appropriate glycemic control [9].

Diabetes self-management plays a central role in the management of type 1 diabetes. In the context of T1D, diabetes self-management behaviors include regular insulin administration, blood glucose monitoring, adherence to dietary recommendations, engagement in physical activity, maintaining a healthy lifestyle, and preventing diabetes related complications [4]. While adults often possess greater disease awareness and experience in managing their condition, children and adolescents may face additional barriers to consistent diabetes self-management, including developmental factors, peer influence, and resistance to parental guidance [3].

Effective Diabetes Self-Management requires not only access to medical care but also the ability to obtain, understand, and apply health information. Health literacy is defined as the capacity to access, comprehend, evaluate, and use health information in order to make appropriate health decisions and maintain well-being [10]. Health literacy is increasingly recognized as a key determinant of successful chronic disease management [10, 11]. Individuals with limited health literacy are more likely to experience difficulties in understanding medical instructions, adhering to treatment regimens, and engaging in effective diabetes improve self-management behaviors. Consequently, low health literacy has been associated with poorer health outcomes, increased hospitalization rates, and higher healthcare costs [12–15]. Previous research has also suggested that patients with type 1 diabetes who have lower levels of health literacy may experience a higher risk of diabetes-related complications [16].

Recent international studies indicate that diabetes self-management education programs can improve diabetes self-management behaviors and glycemic outcomes among adolescents with T1D [17, 18]. Furthermore, contemporary frameworks of adolescent health literacy highlight that young people’s ability to manage chronic conditions is influenced not only by access to health information but also by developmental, cognitive, and digital literacy factors [19–21]. These perspectives suggest that health literacy may play an important role in supporting adolescents to navigate the complex self-management tasks required for effective diabetes control.

Despite the growing body of literature on health literacy in chronic disease management, evidence specifically examining its association with diabetes self-management behaviors — rather than clinical outcomes alone — remains scarce [22]. The majority of existing studies have focused on adult populations, leaving a notable gap in understanding how health literacy shapes diabetes self-management practices among adolescents, who face unique developmental and behavioral challenges. This gap is particularly pronounced in low- and middle-income countries such as Iran, where contextual factors — including health system structure, educational resources, and cultural norms — may modulate this relationship in ways that differ substantially from high-income settings. Addressing this unmet research need is of great importance, as evidence-based insights could inform targeted educational interventions aimed at improving self-management adherence and health outcomes in this vulnerable population [23].

Therefore, the present study aimed to examine the association between health literacy and diabetes self-management behaviors among adolescents aged 10–18 years with type 1 diabetes who have active electronic health records in the SIB system in South Khorasan Province, Iran.

## Materials and methods

### Population and sampling

This cross-sectional analytical study was conducted in 2024 among adolescents aged 10–18 years with type 1 diabetes (T1D) in South Khorasan Province, Iran. Participants were identified through the Integrated Health System (SIB), which serves as the main electronic health record system in the province. Given the limited number of eligible adolescents with T1D in South Khorasan Province, a census sampling method was used rather than random sampling. Since the number of eligible adolescents was small and fully identifiable in the SIB database, no random sampling was performed and all eligible cases were approached. This approach was chosen to include all eligible individuals, maximize sample coverage, increase the precision of estimates, and reduce sampling error and selection bias. According to the SIB records, 149 adolescents met the eligibility criteria. Of these, 9 individuals were not included because they were unavailable or declined to cooperate. Finally, 140 adolescents participated in the study, yielding a response rate of 93.95% (140/149 × 100).

### Data collection

This study was conducted following the official approval from the Ethics Committee for Biomedical Research at Mashhad University of Medical Sciences (Code: IR.MUMS.REC.1403.211). Prior to the commencement of the study, the research objectives were clearly explained to the participants and their families, emphasizing the principles of voluntary participation and confidentiality. Accordingly, written informed consent was obtained from parents or legal guardians, and assent was secured from all participating adolescents.

To enhance rapport and facilitate interaction with the participants, data collection was conducted at healthcare centers by trained public health experts who were native to the region and familiar with the local language and culture. To minimize social desirability bias and ensure the validity of the data, the questionnaires were self-administered in a private and exclusive setting within diabetes clinics. During the completion process, parents and caregivers were asked to wait outside the interview area to prevent any external influence or potential pressure on the adolescents’ responses.

Trained experts remained nearby solely to clarify any potential ambiguities in understanding the questions, without interfering in the response process. Immediately upon completion, each questionnaire was checked for completeness by the researcher. Due to this real-time verification and the immediate resolution of any omissions, there were no missing data in the final dataset; consequently, no data imputation methods were required.

Demographic information—including age, gender, education level, family size, birth order, diabetes duration, insulin injection method, socioeconomic status, and parental occupation and education—was collected using a structured questionnaire.

Health literacy was measured using the Iranian version of the Health Literacy Measure for Adolescents (HELMA) [21], which has demonstrated acceptable reliability and validity (Cronbach’s alpha = 0.77). The questionnaire showed satisfactory content validity (CVI = 0.79, CVR = 0.60) [18]. The Iranian validated version of the Health Literacy Measure for Adolescents (HELMA) was used.

The HELMA questionnaire consists of 44 items that assess adolescent health literacy across eight domains: access, reading, comprehension, evaluation, application, communication, self-efficacy, and calculation. Each item is rated on a 5-point Likert scale ranging from 1 (strongly disagree) to 5 (strongly agree), resulting in a total score range of 44 to 220. Based on the total score, health literacy is categorized into four levels: Inadequate (44–110), Marginal (111–165), Adequate (166–198), and Excellent (199–220). Higher scores indicate better levels of health literacy [22]. In the current study, the internal consistency of the HELMA questionnaire was confirmed with a Cronbach’s alpha of 0.95.

Self-management among adolescents with type 1 diabetes was assessed using the Adolescent Self-Management and Independence in Diabetes (A SMOD) questionnaire [23]. The A SMOD is a validated instrument used to assess diabetes self-management and independence among adolescents with type 1 diabetes. It evaluates the extent and type of diabetes self-management activities performed by adolescents with diabetes across five domains: parental collaboration (10 items), self-care activities (12 items), problem-solving in diabetes (8 items), diabetes-related communication (12 items), and diabetes goals (6 items). Responses are scored on a four-point Likert scale ranging from 1 (never) to 4 (always), with total scores ranging from 48 to 192. Higher scores indicate better diabetes self-management. The validity and reliability of this questionnaire have been confirmed in studies conducted in Iran, with a Cronbach’s alpha of 0.77 [24]. In the present study, the Cronbach’s alpha for the SMOD-A scale was calculated to be 0.85.

### Statistical analysis

Data were analyzed using IBM SPSS Statistics version 26. All variables included in the analysis were complete, with no missing values. The normality of data distribution was assessed using the Shapiro–Wilk test, which indicated that the variables were not normally distributed; therefore, non-parametric statistical tests were applied. Spearman’s rank correlation coefficient was used to assess the association between health literacy and diabetes self-management. Additionally, the Mann–Whitney U test was employed to compare diabetes self-management scores between two independent groups, and the Kruskal–Wallis H test was used for comparisons across more than two groups. A p-value of less than 0.05 was considered statistically significant for all analyses. Effect sizes were interpreted according to Cohen’s (1988) criteria: for eta-squared (η²) and eta-squared for the Kruskal–Wallis H test (η²H), values of 0.01, 0.06, and 0.14 represented small, medium, and large effects, respectively; for correlation coefficients ^®^, values of 0.10, 0.30, and 0.50 represented small, medium, and large effects, respectively. Hierarchical multiple linear regression analysis was performed to examine the independent association between health literacy and diabetes self-management after controlling for relevant demographic and clinical covariates. In the first step, demographic and clinical variables, including age, gender, birth order, duration of diabetes, presence of another diabetic family member, and parental education, were entered into the model. These covariates were selected a priori based on previous literature and their theoretical relevance to adolescents’ health literacy and diabetes self-management. In the second step, the total health literacy score was added to the model to assess its incremental explanatory contribution to the prediction of diabetes self-management beyond demographic and clinical factors. Multicollinearity among the independent variables was assessed using variance inflation factors (VIFs) and tolerance values. All VIF values were below 5 and all tolerance values were above 0.20, indicating no evidence of problematic multicollinearity. The assumptions of linear regression, including linearity, normality of residuals, homoscedasticity, and independence of errors, were evaluated using residual plots, normal probability plots, and the Durbin–Watson statistic. The overall model fit was assessed using *R*^2^, adjusted *R*^2^, *F*-statistics, and the change in *R*^2^ (Δ*R*^2^) between regression steps.

## Results

A total of 140 adolescents with type 1 diabetes participated in the study. The mean age of the participants was 13.9 ± 2.3 years. The mean health literacy score was 135.79 ± 28.4, which according to HELMA classification falls within the marginal health literacy range. The mean diabetes self-management score was 133.36 ± 20.22.

### Demographic and clinical characteristics of participants

The majority of participants were aged 13–15 years (51.4%), while those in the 10–12 and 16–18 age groups each accounted for 24.3% of the sample. Over half of the participants were female (55.0%). Regarding clinical characteristics, 60.7% had a diabetes duration of less than three years, and 24.3% reported having another family member with diabetes. In terms of family background, most adolescents were the first or second child (73.6%). Regarding parental education, 61.4% of fathers and 75.0% of mothers had attained an education level above a high school diploma. Detailed demographic and clinical characteristics are presented in Table 1.

**Table 1:** Baseline demographic and clinical characteristics of participants

Characteristics of Adolescents with Type 1 Diabetes		Number (*n*)	Percentage (%)
Age	10–12	34	24.3%
	13–15	72	51.4%
	16–18	34	24.3%
Gender	Female	77	55.0%
	Male	63	45.0%
Birth Order	First and second	103	73.6%
	Third and fourth	37	26.4%
Diabetes History	Less than 3 years	85	60.7%
	More than 3 years	55	39.3%
Another Diabetic Individual	Yes	34	24.3%
	No	106	75.7%
Father’s Education	Below Diploma	54	38.6%
	Above Diploma	86	61.4%
Mother’s Education	Below Diploma	35	25.0%
	Above Diploma	105	75.0**%**

### Factors associated with diabetes self-management

In this study, the highest mean Diabetes Self-Management score was observed among adolescents aged 16–18 years (144.76 ± 18.45; range: 48–192), while the lowest score was found in those aged 10–12 years (120.08 ± 13.48), with this difference being statistically significant (*p* < 0.001). Regarding birth order, adolescents in the first and second positions had a higher mean Diabetes Self-Management score (135.33 ± 21.06) compared to those in the third and fourth positions (127.86 ± 16.72); however, this difference was not statistically significant (*p* = 0.087). The mean Diabetes Self-Management scores for females (134.09 ± 18.63) and males (132.47 ± 22.12) did not show a statistically significant difference (*p* = 0.559).

Additionally, adolescents diagnosed with diabetes for more than three years had a higher mean Diabetes Self-Management score (143.16 ± 18.49) compared to those diagnosed for less than three years (*p* < 0.001). The presence of another diabetic individual in the family was associated with a higher mean Diabetes Self-Management score (146.08 ± 20.12), which was statistically significant (*p* < 0.001). Furthermore, higher levels of both maternal and paternal education were significantly associated with increased Diabetes Self-Management scores in adolescents (*p* = 0.002 and *p* = 0.026, respectively). Effect size analysis showed small to moderate effects for father’s education (*r* = 0.19) and mother’s education (*r* = 0.27). A relatively large effect was observed for history of diabetes (*r* = 0.38) and the presence of another diabetic family member (*r* = 0.35). Age showed a large effect on Diabetes Self-Management (η²H = 0.16), while gender (*r* = 0.05) and birth order (*r* = 0.14) demonstrated small effects (Table 2).

**Table 2:** Diabetes self-management scores according to demographic and clinical characteristics of adolescents with type 1 diabetes

Characteristics of Adolescents with Type 1 Diabetes		Diabetes Self-Management Score, Mean ± SD	Effect size	*p*
Age	10–12	120.08 ± 13.48	η²H = 0.16	< 0.001**
	13–15	134.25 ± 20.25		
	16–18	144.76 ± 18.45		
Gender	Female	134.09 ± 18.63	*r* = 0.05	0.559*
	Male	132.47 ± 22.12		
Birth Order	First and second	135.33 ± 21.06	*r* = 0.14	0.087*
	Third and fourth	127.86 ± 16.72		
Diabetes History	Less than 3 years	127.02 ± 18.79	*r* = 0.38	< 0.001*
	More than 3 years	143.16 ± 18.49		
Another Diabetic Individual	Yes	146.08 ± 20.12	*r* = 0.35	< 0.001*
	No	129.28 ± 18.56		
Father’s Education	Below Diploma	128.51 ± 17.90	*r* = 0.19	0.026*
	Above Diploma	136.40 ± 21.07		
Mother’s Education	Below Diploma	124.02 ± 14.48	*r* = 0.27	0.002*
	Above Diploma	136.47 ± 20.94		

Effect sizes were interpreted according to Cohen’s criteria

Effect sizes were calculated as r = |Z|/√N for Mann–Whitney tests and η²H = (H − k + 1)/ (N − k) for Kruskal–Wallis tests. Significance level was set at *p* < 0.05

^*^Mann–Whitney U test; ^**^Kruskal–Wallis test. Values are presented as mean ± SD

### Demographic variables and health literacy levels

Adolescents aged 16–18 demonstrated the highest proportion of adequate health literacy (64.7%), whereas only 2.9% of those aged 10–12 achieved adequate health literacy. The greatest proportion of inadequate health literacy was observed in the 13–15 age group (13.9%) (*p* < 0.001). Regarding gender, 39.0% of females and 36.5% of males had adequate health literacy (*p* = 0.87). First- and second-born adolescents showed a higher proportion of adequate health literacy (43.7%) compared with third- and fourth-born adolescents (*p* = 0.002). Adolescents with more than three years of diabetes history exhibited a higher proportion of adequate health literacy (61.8%) than those with shorter disease duration (*p* < 0.001). Having another diabetic family member was also associated with a greater proportion of adequate health literacy (73.5%) (*p* < 0.001). In terms of parental education, adolescents whose mothers had education levels above high school demonstrated 45.7% adequate and 14.3% inadequate health literacy (*p* = 0.001). Similarly, adolescents whose fathers had education levels above high school reported a higher proportion of adequate health literacy (45.3%) compared with those whose fathers had lower educational attainment (*p* = 0.016) (Table 3).

**Table 3:** Association between demographic variables and health literacy levels among adolescents with type 1 diabetes

Characteristics of Adolescents with Type 1 Diabetes	category	Health Literacy Levels					*p*-value
		Inadequate health literacy *n* (%)	Marginal health literacy *n* (%)	Adequate health literacy *n* (%)	Excellent health literacy *n* (%)	Total *n*	
Age	10–12	9 (26.5)	24 (70.6)	1 (2.9)	0 (0.0)	34	< 0.001*
	13–15	10 (13.9)	28 (38.9)	30 (41.7)	4 (5.6)	72	
	16–18	3 (8.8)	9 (26.5)	22 (64.7)	0 (0.0)	34	
Gender	Female	12 (15.6)	33 (42.9)	30 (39.0)	2 (2.6)	77	0.87**
	Male	10 (15.9)	28 (44.4)	23 (36.5)	2 (3.2)	63	
Birth Order	First and second	15 (14.6)	40 (38.8)	45 (43.7)	3 (2.9)	103	0.002**
	Third and fourth	7 (18.9)	21 (56.8)	8 (21.6)	1 (2.7)	37	
Diabetes History	Less than 3 years	20 (23.5)	46 (54.1)	19 (22.4)	0 (0.0)	85	< 0.001**
	More than 3 years	2 (3.6)	15 (27.3)	34 (61.8)	4 (7.3)	55	
Another Diabetic Individual	Yes	2 (5.9)	6 (17.6)	25 (73.5)	1 (2.9)	34	< 0.001**
	No	20 (18.9)	55 (51.9)	28 (26.4)	3 (2.8)	106	
Father’s Education	Below Diploma	9 (16.7)	31 (57.4)	14 (25.9)	0 (0.0)	54	0.016**
	Above Diploma	13 (15.1)	30 (34.9)	39 (45.3)	4 (4.7)	86	
Mother’s Education	Below Diploma	7 (20.0)	23 (65.7)	5 (14.3)	0 (0.0)	35	0.001**
	Above Diploma	15 (14.3)	38 (36.2)	48 (45.7)	4 (3.8)	105	

Values are presented as *n* (%). Percentages are calculated within each demographic subgroup (row percentages), rather than as percentages of the total sample (*n* = 140). Accordingly, percentages within each row sum to approximately 100%. This approach follows standard practice for cross-tabulation analyses

^*^Kruskal–Wallis test, ^**^Mann–Whitney U test

### Health literacy dimensions and diabetes self-management

The Spearman’s rank correlation analysis between different dimensions of health literacy and Diabetes Self-Management in adolescents with type 1 diabetes showed that all dimensions of health literacy had significant associations with Diabetes Self-Management (*p* < 0.01). Self-efficacy demonstrated the highest correlation with Diabetes Self-Management (Spearman’s rho = 0.794), followed by calculation (rho = 0.771) and evaluation (rho = 0.769). Other dimensions also showed significant correlations, including comprehension (rho = 0.678), communication (rho = 0.669), and access (rho = 0.644). Health information application (rho = 0.475) and reading (rho = 0.538) had the lowest correlations with Diabetes Self-Management (Table 4).

**Table 4:** Correlation between health literacy dimensions and diabetes self-management

Health Literacy Dimensions	Spearman’s rho (*ρ*)	*p*-value
Access	0.644	< 0.001
Reading	0.538	< 0.001
Comprehension	0.678	< 0.001
Evaluation	0.769	< 0.001
Application	0.475	< 0.001
Communication	0.669	< 0.001
Self-Efficacy	0.794	< 0.001
Calculation	0.771	< 0.001
Total Health Literacy Score	0.812	< 0.001

### Independent contribution of health literacy to diabetes self-management

To evaluate the independent contribution of health literacy to Diabetes Self-Management, a two-step hierarchical multiple regression analysis was performed. In Step 1, demographic and clinical variables, including age, gender, birth order, duration of diabetes, presence of another diabetic family member, and parental education, were entered into the model. This model explained 34.1% of the variance in Diabetes Self-Management (R² = 0.341, adjusted R² = 0.306, F(7,132) = 9.77, *p* < 0.001).

In Step 2, the HELMA total health literacy score was added to the model. The final model explained 70.1% of the variance in Diabetes Self-Management (R² = 0.701, adjusted R² = 0.683, F(8,131) = 38.39, *p* < 0.001). The addition of health literacy resulted in a significant increase in explained variance (ΔR² = 0.360), This indicates that health literacy alone accounted for an additional 36% of the variance in Diabetes Self-Management.In the final model, health literacy was the strongest factor associated with Diabetes Self-Management (β = 0.76, *p* < 0.001), followed by age (β = 0.13, *p* = 0.019). The coefficient for “another diabetic in family” was statistically significant in Step 1 (B = − 11.96, *p* = 0.001) but became non-significant after adding health literacy in Step 2 (B = − 2.07, *p* = 0.405).

This attenuation suggests that the association between family history of diabetes and Diabetes Self-Management may be partly explained by health literacy and other adjusted covariates. However, given the cross-sectional design, this finding should be interpreted as an adjusted association rather than evidence of mediation or causality (Table 5).

**Table 5:** Hierarchical multiple regression analysis predicting diabetes self-management among adolescents with type 1 diabetes

Predictor	B (SE)	β	95% CI	t	*p*
Step 1					
(Constant)	108.94 (13.50)	—	[82.23, 135.65]	8.07	< 0.001
Gender	2.89 (2.97)	0.07	[-2.97, 8.76]	0.98	0.331
Another diabetic in family	-11.96 (3.48)	-0.25	[-18.84, -5.08]	-3.44	0.001
Age	8.82 (2.27)	0.31	[4.33, 13.31]	3.89	< 0.001
Birth order	0.61 (3.41)	0.01	[-6.14, 7.36]	0.18	0.858
Duration of diabetes	10.08 (3.18)	0.24	[3.79, 16.37]	3.17	0.002
Mother’s Education	5.79 (4.93)	0.12	[-3.96, 15.54]	1.18	0.242
Father’s Education	-0.84 (4.32)	-0.02	[-9.38, 7.71]	-0.19	0.846
Step 2					
(Constant)	50.73 (10.24)	—	[30.47, 70.99]	4.95	< 0.001
Gender	-0.37 (2.02)	-0.01	[-4.37, 3.63]	-0.18	0.854
Another diabetic in family	-2.07 (2.48)	-0.04	[-6.98, 2.84]	-0.84	0.405
Age	3.78 (1.59)	0.13	[0.64, 6.91]	2.38	0.019
Birth order	1.66 (2.31)	0.04	[-2.90, 6.23]	0.72	0.472
Duration of diabetes	-0.54 (2.31)	-0.01	[-5.11, 4.03]	-0.23	0.816
Mother’s Education	5.82 (3.33)	0.13	[-0.78, 12.41]	1.75	0.083
Father’s Education	-3.34 (2.93)	-0.08	[-9.13, 2.46]	-1.14	0.257
Health literacy (HELMA total)	0.54 (0.04)	0.76	[0.45, 0.62]	12.55	< 0.001

Step 1 included demographic and clinical variables (Gender, Another diabetic in family, Age, Birth order, Duration of diabetes, Mother’s Education, Father’s Education). Step 2 included the aforementioned variables plus the HELMA total health literacy score. B, unstandardized regression coefficient; SE, standard error; β, standardized regression coefficient; CI, 95% confidence interval. Model fit indices: Model 1: R² = 0.341, Adjusted R² = 0.306; F(7,132) = 9.77, *p* < 0.001, Model 2: R² = 0.701, Adjusted R² = 0.683; F(8,131) = 38.39, *p* < 0.001

Multicollinearity diagnostics indicated no problematic multicollinearity, with all VIF values < 5 and tolerance values > 0.20

### Health literacy levels and diabetes self-management

Diabetes Self-Management scores differed significantly across health literacy levels among adolescents with type 1 diabetes (Kruskal–Wallis H(3) = 102.662, *p* < 0.001, η²H = 0.717; Table 6). Adolescents with inadequate health literacy had the lowest Diabetes Self-Management score (M = 112.45, SD = 9.66; *n* = 22), followed by those with marginal health literacy (M = 120.96, SD = 9.19; *n* = 61). The highest mean Diabetes Self-Management score was observed in adolescents with adequate health literacy (M = 155.41, SD = 9.18; *n* = 53). Participants with excellent health literacy had a mean Diabetes Self-Management score of 145.25 (SD = 4.99; *n* = 4); however, because this subgroup was very small, comparisons involving this category should be interpreted cautiously.

**Table 6:** Mean scores of diabetes self-management by health literacy levels

Health Literacy Levels	Mean of Diabetes Self-Management (M)	*n* (%)	Standard Deviation (SD)	*p*-value
Inadequate Health Literacy	112.45	22 (15.7)	9.66	< 0.001*
Marginal Health Literacy	120.96	61 (43.6)	9.19	
Adequate Health Literacy	155.41	53 (37.9)	9.18	
Excellent Health Literacy	145.25	4 (2.9)	4.99	

Kruskal–Wallis H(3) = 102.662, *p* < 0.001, $$\upeta_{H}^{2}=0.717$$

### Domains of diabetes self-management

**Fig. 1:**
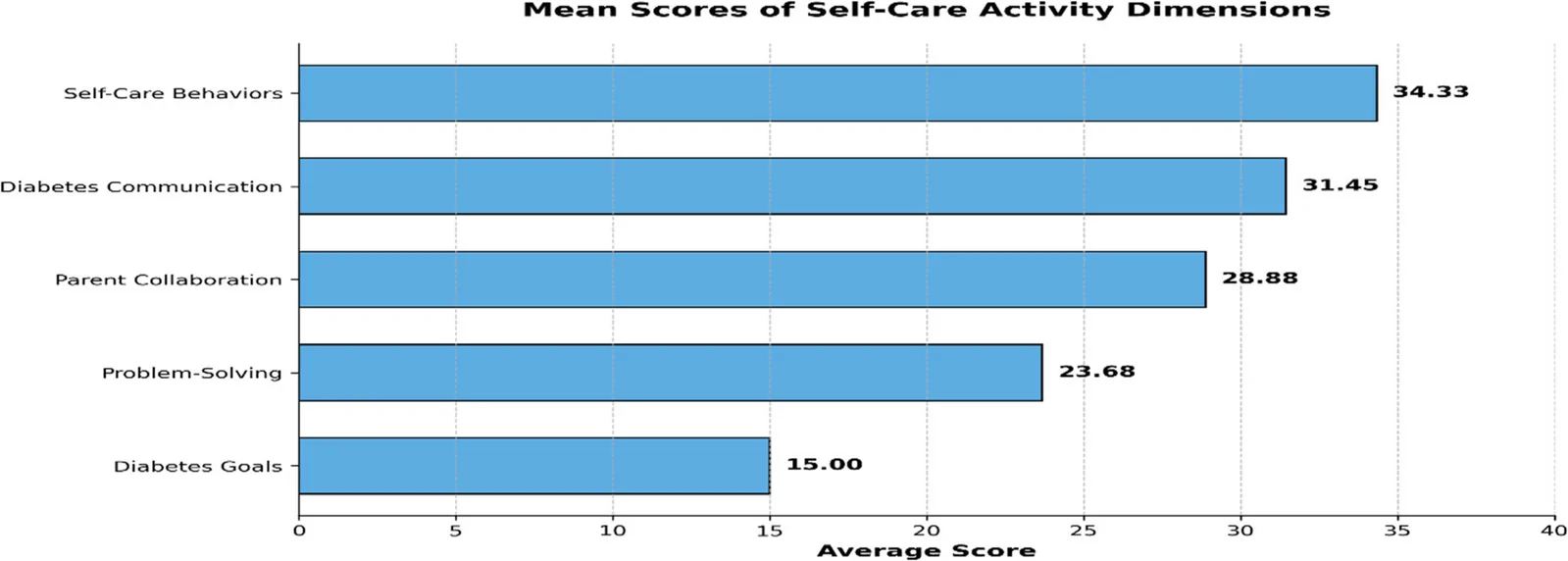
Mean scores of self-reported self-management among adolescents with type 1 diabetes

The self-reported self-management activities by adolescents are presented in Fig. 1. The highest reported activities were related to Diabetes Self-Management, with a mean score of 34.33, while the lowest mean score was for diabetes goals, with a score of 15. Diabetes communication (31.45), parental involvement (28.88), and problem-solving (23.68) were also among the next highest-ranked activities.

## Discussion

The present findings indicate a significant positive association between health literacy and Diabetes Self-Management among adolescents with type 1 diabetes. Adolescents with higher health literacy reported better Diabetes Self-Management, which is consistent with previous evidence linking health literacy to diabetes self-management outcomes [25, 26]. This association suggests that health literacy may be an important modifiable factor in adolescent diabetes care, particularly because effective self-management requires the ability to understand, evaluate, and apply complex health information in daily life. Beyond mere knowledge acquisition, higher health literacy may improve communication with healthcare providers, enhance problem-solving capacities, and strengthen confidence in daily management decisions—skills that are particularly critical during adolescence as self-management responsibility gradually shifts from caregivers to the adolescent. Hierarchical regression analysis indicated that health literacy remained a significant independent correlate of Diabetes Self-Managementa fter controlling for demographic and clinical variables, suggesting that its contribution extends beyond age, gender, and disease duration. Because the study design was cross-sectional, these findings reflect associations only; causal inferences cannot be drawn. Longitudinal and interventional studies are needed to determine whether targeted health literacy interventions lead to sustained improvements in Diabetes Self-Management. Age and parental education were also related to Diabetes Self-Management. Older adolescents tended to demonstrate better Diabetes Self-Management, possibly reflecting greater autonomy, accumulated disease-management experience, and improved capacity to apply health information, although increased independence does not always translate to optimal self-management, underscoring the value of age-appropriate support. Higher maternal and paternal education was similarly linked to better adolescent Diabetes Self-Management [27, 28], likely because educated parents are better equipped to access health information, understand management recommendations, and communicate effectively with healthcare teams. These findings collectively support incorporating both age-sensitive approaches and parental involvement into diabetes education programs. Having another family member with diabetes also appeared relevant to Diabetes Self-Management. Shared lived experience within the family may facilitate informal learning, emotional modeling, and practical support for disease management routines. The observed change in the regression coefficient for this variable after health literacy was entered into the model may suggest partial overlap in their explanatory roles, although this interpretation requires caution given shared variance among predictors. Among the dimensions of health literacy, self-efficacy, calculation, and evaluation showed the strongest correlations with Diabetes Self-Management, a finding with clear clinical relevance. Effective diabetes management demands accurate interpretation of health information, insulin and carbohydrate calculations, evaluation of glucose trends, and real-time decision-making [29]. Diabetes education programs should therefore actively strengthen these specific competencies rather than focusing solely on information delivery. A slight decrease in Diabetes Self-Management scores was observed in the “Excellent” health literacy subgroup compared with the “Adequate” group. This finding should be interpreted with considerable caution: the “Excellent” subgroup comprised only four participants, and the observed decline is most likely a statistical artifact reflecting sampling instability in this very small subgroup rather than a true behavioral pattern. Larger, more balanced samples are needed before any conclusions can be drawn regarding this subgroup. Health literacy alone may not fully account for Diabetes Self-Management variability. Social support, psychological empowerment, stress, economic factors, family relationships, peer interactions, and access to healthcare resources all contribute to adolescent self-management capacity [30, 31]. Future interventions should therefore integrate health literacy enhancement with family-based support, behavioral counseling, and adolescent-centered educational strategies. From a practical standpoint, routine screening for limited health literacy in diabetes clinics can help identify at-risk adolescents. Educational materials should be literacy-appropriate and incorporate clear language, visual aids, interactive methods, and practical exercises, with emphasis on numeracy, evaluation, self-efficacy, and communication skills. Involving parents and family members in educational programs can further reinforce a supportive home environment for sustainable diabetes self-management.

### Limitations

This study has several limitations. First, the cross-sectional design precludes any causal inference regarding the relationship between health literacy and Diabetes Self-Management. Therefore, the observed associations should not be interpreted as evidence that higher health literacy directly leads to better Diabetes Self-Management. Second, both health literacy and Diabetes Self-Management were assessed using self-reported questionnaires, which may be affected by recall bias, social desirability bias, and common method bias. Third, objective clinical outcomes such as HbA1c, frequency of hypoglycemia, or diabetes-related hospitalizations were not available; therefore, the relationship between health literacy, Diabetes Self-Management, and actual glycemic control could not be examined. Fourth, the study was conducted in a single province in eastern Iran, which may limit the generalizability of the findings to adolescents with type 1 diabetes in other regions or healthcare settings. Additionally, the use of the SIB registry as the sampling frame may have excluded eligible adolescents who did not have active electronic health records, which should be considered when interpreting the generalizability of the findings. Finally, the “Excellent” health literacy subgroup included only four participants, reducing the reliability of subgroup comparisons and requiring cautious interpretation of findings related to this category. Additionally, a potential conceptual overlap between the HELMA and A-SMOD instruments may have modestly inflated the observed association between health literacy and Diabetes Self-Management. Future longitudinal studies using larger and more diverse samples, multi-method assessments, and objective clinical indicators are recommended.

## Conclusion

This study demonstrated a strong positive association between health literacy and Diabetes Self-Management in adolescents with type 1 diabetes in South Khorasan Province, Iran. Overall health literacy was at a marginal level, underscoring a critical gap in this population. Among the eight HELMA domains, self-efficacy, calculation, and evaluation showed the strongest correlations with Diabetes Self-Management, identifying these as priority targets for intervention. Older adolescents and those with more educated parents or a family member with diabetes demonstrated significantly better Diabetes Self-Management and health literacy, highlighting the role of family and social context. These findings call for structured, literacy-based educational interventions — particularly targeting numeracy, critical evaluation, and self-efficacy — delivered in collaboration with families. Future longitudinal studies with objective outcomes (e.g., HbA1c) are needed to establish causality and evaluate intervention effectiveness.

## Data Availability

The datasets generated and/or analyzed during the current study are available from the corresponding author upon reasonable request.
